# Facile Solvothermal Synthesis and Gas Sensitivity of Graphene/WO_3_ Nanocomposites

**DOI:** 10.3390/ma7064587

**Published:** 2014-06-17

**Authors:** Yanghai Gui, Junhua Yuan, Weiming Wang, Jianbo Zhao, Junfeng Tian, Bing Xie

**Affiliations:** 1 Department of Material and Chemical Engineering, Zhengzhou University of Light Industry, Zhengzhou 450002, Henan, China; E-Mails: wmwang@163.com (W.W.); zjianb1124@163.com (J.Z.); tjf0101@zzuli.edu.cn (J.T.); xiebing@zzuli.edu.cn (B.X.); 2 College of Life Sciences and Chemistry, Zhejiang Normal University, Jinhua 321004, Zhejiang, China; E-Mail: jhyuan@zjnu.cn

**Keywords:** WO_3_, graphene/WO_3_, solvothermal, gas sensor

## Abstract

Graphene has attracted enormous attention owing to its extraordinary properties, while graphene-based nanocomposites hold promise for many applications. In this paper, we present a two-step exploitation method for preparation of graphene oxides and a facile solvothermal route for preparation of few-layer graphene nanosheets and graphene/WO_3_ nanocomposites in an ethanol-distilled water medium. The as-synthesized samples were characterized by using field emission scanning electron microscopy (FE-SEM), high-resolution transmission electron microscopy (HRTEM), ultraviolet-visible (UV-vis) spectroscopy, Raman spectroscopy, X-ray diffraction (XRD), thermogravimetric-differential thermal analysis (TG-DTA) and gas-sensing test. The resistivity of the thick-film gas sensors based on sandwich-like graphene/WO_3_ nanocomposites can be controlled by varying the amount of graphene in the composites. Graphene/WO_3_ nanocomposites with graphene content higher than 1% show fast response, high selectivity and fine sensitivity to NO_x_.

## 1. Introduction

Graphene, whose carbon atoms are arranged in a closely packed honeycomb two-dimensional lattice, has attracted enormous attention owing to its extraordinary electrical, thermal and mechanical properties [1,2]. Because of their extraordinary properties, graphene and graphene-based composites have many potential applications in catalysis, fuel cells, sensors and lithium ion batteries [3,4,5,6,7,8,9]. As we all know, graphene also has applications in chemical sensing because of its high surface area. A few attempts have been made to study the gas sensitivity of graphene-based gas sensors [6,10,11,12]. Recent studies have shown that a thin film of graphene has a high sensitivity to NO_x_, H_2_ and H_2_S, but few works have been reported on tungsten oxide and graphene hybrid nanocomposite thick-film gas sensors [13,14].

WO_3_ is a well-known semiconductor material used for gas sensors to monitor toxic and explosive gases (e.g., NO_x_, H_2_S, CO and NH_3_) [15,16,17,18,19,20]. However, the range of application of WO_3_ gas sensors is limited by poor selectivity, long response time, low sensitivity and high resistivity. Although methods, such as reduction of grain size, use of novel synthesis morphology, addition of dopants, and use of mixed sensing materials, have been adopted to enhance the sensing properties of WO_3_, fewer contributions have been made to reducing the high resistivity of WO_3_-based sensors [21,22].

In this paper, resistivity-controlled thick-film sensors based on few-layer graphene nanosheets and graphene/WO_3_ nanocomposites were prepared by a facile solvothermal method in an ethanol-distilled water medium. Material characterizations of the prepared samples were carried out by FE-SEM, HRTEM, UV-vis spectroscopy, Raman spectroscopy, XRD and TG-DTA. Gas-sensing properties of the sensors based on pure WO_3_, few-layer graphene nanosheets, and graphene/WO_3_ nanocomposites were investigated by using a HW-30A gas-sensing measurement system.

## 2. Results and Discussion

**Figure 1:**
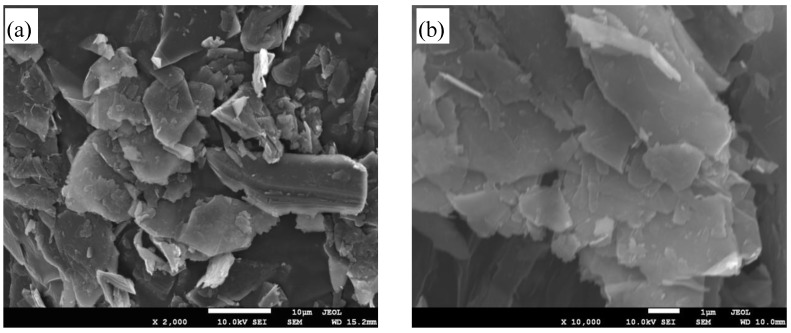
SEM images of (**a**) graphite prepared from nature graphite by ultrasonication for 30 min and (**b**) exploited graphite (EG).

Morphologies of the first exploited graphite (EG) and graphite prepared from natural graphite by ultrasonication for 30 min are shown in Figure 1. Comparing EG (Figure 1a) with graphite (Figure 1b), it is clear that the bulk graphite was effectively exploited to graphite nanosheets after the first step of exfoliation of graphene. The exploitation mechanism may be the hydrogen peroxide and ammonium persulfate solution dispersing into the interspace of graphite layers through the ultrasonication process. Under microwave radiation, the hydrogen peroxide decomposes and intercalates into the interlayer with ammonium persulfate. Subsequently, the residual hydrogen peroxide and ammonium persulfate decompose as the reaction proceeds further. The process was exothermic and caused a rapid expansion and the exploited bulk graphite rapidly formed the graphite flake with nanostructure, accompanied with lightening.

**Figure 2:**
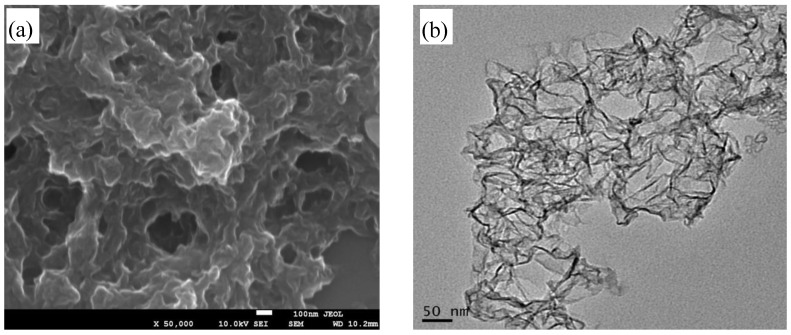
Morphology of FG prepared via facile solvothermal route: (**a**) FE-SEM image; and (**b**) TEM image.

Figure 2a shows TEM image of the as-synthesized few-layer graphene nanosheets (FG). Surfaces of the FG sheets are rough within nanoscale with some wrinkles and agglomeration, which may be attributed to the residual oxygen-containing functional groups (e.g., –COOH, –OH and –COH). Figure 2b presents TEM image of the FG nanosheet. It is evident that FG nanosheets are crumpled, folded sheets which are entangled together. The phenomenon of crumpling and scrolling is part of the intrinsic nature of graphene nanosheets, which is resulted from the fact that the two-dimensional membrane structure becomes thermodynamically stable via bending.

**Figure 3:**
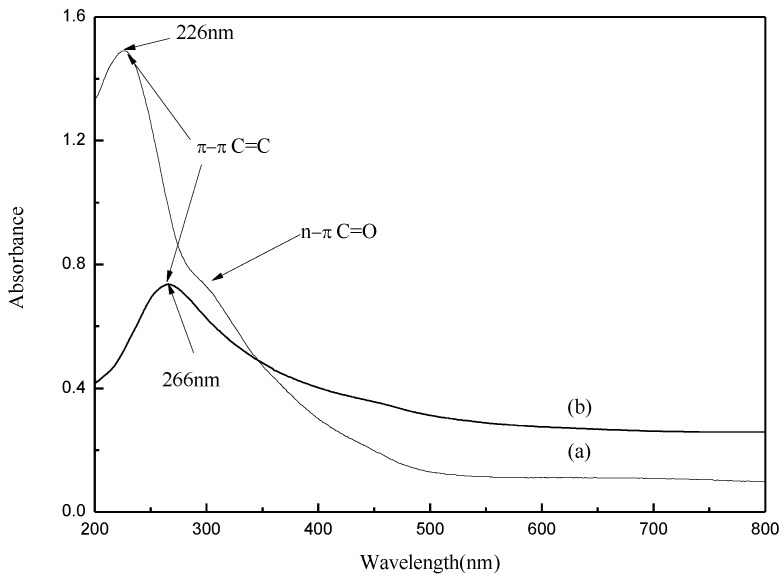
UV-vis spectra of (a) the FGO prepared via two step exploitation of graphite and (b) FG.

Figure 3 shows UV-vis absorption spectra of the as-synthesized few-layer graphene oxide (FGO) and FG. For the UV-vis measurements, the samples were dispersed in ethanol by ultrasonication. The UV-vis spectrum was employed to monitor the restoration of conjugated C=C bonds of FGO. It can be seen that the FGO shows a strong absorption peak at 226 nm (Figure 3a). The values indicate that a large number of double-bond conjugations (C=C and C=O) exist in FGO. FG shows an absorption peak at 266 nm, which is generally regarded as the excitation of the π-plasmon of the graphitic structure (Figure 3b). There is no obvious absorption peak at 226 nm in FG, indicating that the conjugated C=C bonds were effectively restored by the solvothermal reduction.

**Figure 4:**
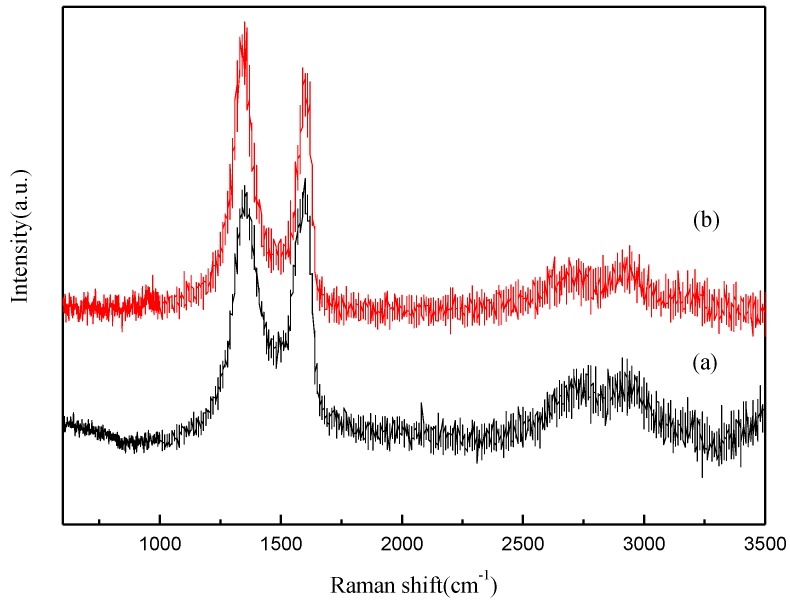
Raman spectra of the samples: (a) FGO; (b) FG.

Raman spectroscopy is a nondestructive and efficient approach to characterize graphitic materials, especially for determining ordered and disordered crystal structures of graphene. The Raman spectra of FGO and FG are shown in Figure 4. Both FGO and FG display two prominent peaks (D band and G band). The peak at 1380 cm^−1^, labeled as the D band, corresponds to breathing modes of rings or K-point phonons of A_1g_ symmetry, whereas the G band at 1580 cm^−1^ corresponds to an E_2g_ mode of graphite and is related to the vibration of sp^2^ bonded carbon atoms in a two-dimensional hexagonal lattice. The ratio of the intensity of the G band to that of the D band is related to the in-plane crystallite size, *L*_a_. The in-plane crystallite size of FGO and FG, calculated by using the relationship *L*_a_ (nm) = 4.4 (I(G)/I(D)), about 4.51 and 3.7 nm, respectively, for which the corresponding I(G)/I(D) ratios are 1.0242 and 0.8315, respectively [23]. Compared with FGO, the I(G)/I(D) of FG decrease indicates the removal of oxygen-containing functional groups in FGO. The disorder-induced combination mode band (G + D) can be seen along with the weak 2D band at 2930 cm^−1^, making it conceivable that the sample contained highly disordered and randomly arranged graphene sheets.

**Figure 5:**
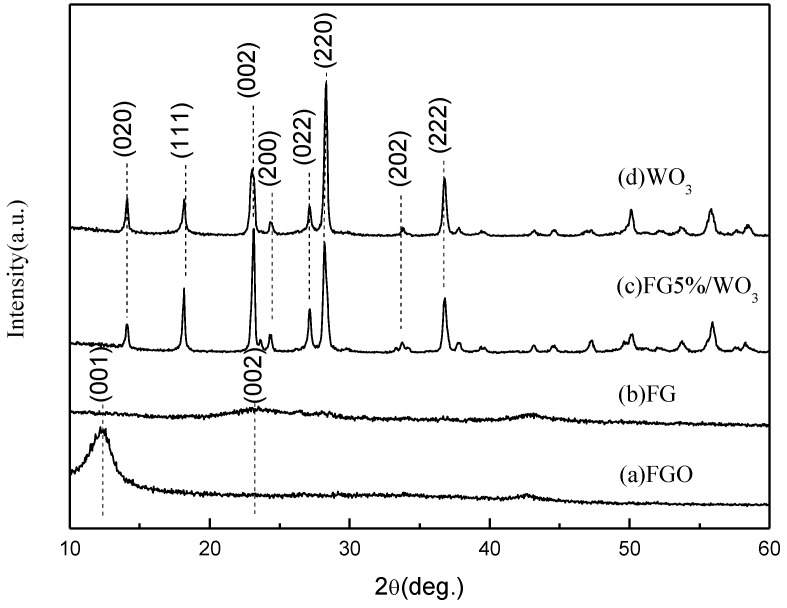
X-ray diffraction patterns of the samples: (a) GO; (b) FG; (c) FG5%/WO_3_ nanocomposites and (d) WO_3_.

XRD measurements were employed to investigate phase and structure of the synthesized samples. As shown in Figure 5, the XRD pattern of the as-synthesized FGO (Figure 5a) shows a peak at 2θ = 12.201°, corresponding to the (001) reflection of graphite oxide, and the interlayer spacing (0.73 nm) is much larger than that of natural graphite (about 0.34 nm) owing to the introduction of oxygen-containing functional groups on the surface of the graphite sheets [24]. From Figure 5b, the broad diffraction peak at 2θ = 23.45° is the (002) reflection of FG, which is different from the (002) reflection of graphite at 2θ = 26.44° [25]. This difference is due to the residual functional groups present between the graphene layers and the short-range order of the graphene sheets along the stacking direction. Compared with graphite oxide (GO), the (001) reflection of FG has disappeared, suggesting the removal of the oxygen-containing functional groups on the FG surfaces. As shown in Figure 5c, all the diffraction peaks of the FG/WO_3_ nanocomposites can be indexed to hexagonal WO_3_(H_2_O)_0.33_ (JCPDS 87-1203), which is the same as the WO_3_ sample (Figure 5d), and no characteristic peak of graphite was observed, suggesting that the restacking of the as-reduced graphene sheets was effectively prevented. Compared with the WO_3_ sample, the most intense peak of FG/WO_3_ nanocomposites was changed from (220) to (002), which indicated that the preferred growth direction of WO_3_ was changed.

**Figure 6:**
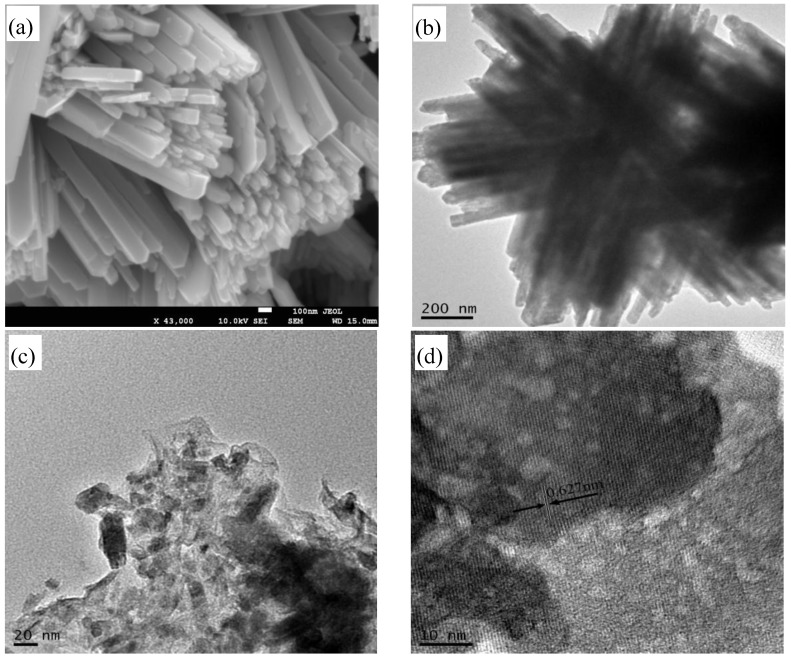
Morphologies of samples: (**a**) SEM images of WO_3_ synthesized via Solvothermal; (**b**) TEM images of WO_3_; (**c**,**d**) TEM images of FG (5%)/WO_3_ nanocomposites.

Morphologies of WO_3_ and the FG (5%)/WO_3_ nanocomposites were further analyzed by FE-SEM and HRTEM. As shown in Figure 6a, the as-synthesized WO_3_ sample exhibits unordered bundles with uniform strip size. TEM investigation can identify the bundled feature, giving evidence that each one-dimensional nanostructure bundle consists of a nanostrip with a thickness of about 100 nm, as shown in Figure 6b. Figure 6c,d show the TEM images of FG(5%)/WO_3_ nanocomposites. As displayed in Figure 6c, WO_3_ particles distributed randomly on the FG surface with different morphology are easily observed. The morphology change of WO_3_ indicates that the addition of FG has a significant effect on the WO_3_ particle growth, which is consistent with the XRD result, *i.e.*, the preferred growth direction of WO_3_ was changed. The high-resolution TEM image of another area of the sample, given in Figure 6b, clearly shows the uniform lattice fringes. The spacing between the lattice fringes is about 0.627 nm, which can be indexed to the (020) plane of the hexagonal WO_3_(H_2_O)_0.33_. This is evidence for the existence of a sandwich-like structure between graphene and WO_3_. It is believed that both oxygen-containing defect sites and pristine regions of the GO favorably interact with W^6+^ via van der Waals interactions and chemisorptions, respectively. In addition, oxygen functional groups located at the surface of GO can act as anchor sites and effectively hinder diffusion, recrystallization, and growth of WO_3_ grains. The two morphologies of FG/WO_3_ nanocomposites may be formed via isolated growth of WO_3_ crystal species at the surface of GO and *in situ* growth of WO_3_ crystal species on the surface of GO, respectively.

**Figure 7:**
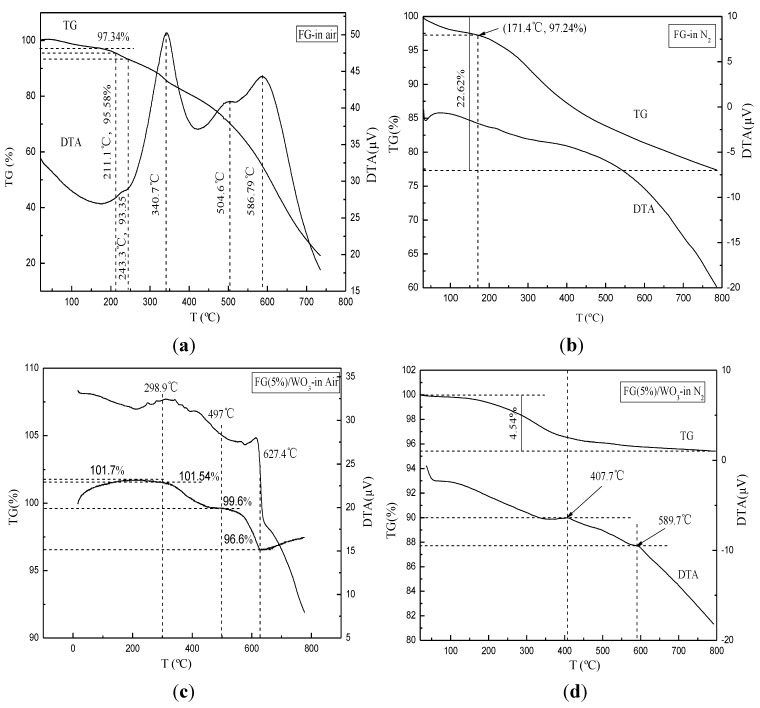
TG-DTA curves of the FG and FG (5%)/WO_3_ nanocomposites: (**a**) FG in air; (**b**) FG (5%)/WO_3_ nanocomposites in air; (**c**) FG in N_2_; (**d**) FG (5%)/WO_3_ nanocomposites in N_2_.

Thermal properties and composition of the FG and FG (5%)/WO_3_ nanocomposites synthesized via the solvothermal method were characterized by TG-DTA, which was performed in air and N_2_ atmospheres with a heating rate of 15 °C·min^−1^. As shown in Figure 7a, with increasing temperature, the FG synthesized via the solvothermal method shows a gradual weight loss during the whole process, confirming that the thermal stability of the chemically derived graphene is much lower than that of the bulk graphite powders [25]. The weight loss is usually attributed to the loss of the residual (or absorbed) solvent and the decomposition of residual organic functional groups on FG. After that, the weight loss occurs above 420 °C, which can be assigned to the oxidation of graphene in air. Correspondingly, the DTA curve shows a weak exothermal peak centered at 504 °C and a strong exothermal peak centered at 586 °C, which can be assigned to the oxidation of few-layer graphene (single, double, and triple layer) and many-layer graphene in air, respectively. As shown in Figure 7b, with increasing temperature, FG shows a gradual weight loss, which indicates that the decomposition of residual organic functional groups on graphene occurs throughout the whole thermal analysis process. It was obvious that the weight loss in N_2_ atmosphere is significantly lower than that in air atmosphere especially at temperatures higher than 450 °C, which should result from the combustion of the carbon skeleton of graphene happened above 420 °C in air.

From Figure 7c, it can be seen that the FG (5%)/WO_3_ nanocomposites synthesized via the solvothermal method shows two abrupt weight losses occurring between 300 and 630 °C in air atmosphere. Correspondingly, the DTA curve shows an endothermic peak and an exothermal peak, which can be assigned to the loss of chemically bonded water and oxidation of graphene in air, respectively. As shown in Figure 7d, the TG curve of FG (5%)/WO_3_ nanocomposites performed in N_2_ atmosphere exhibits a continuous weight loss, which can be assigned to the loss of chemically bonded water.

**Figure 8:**
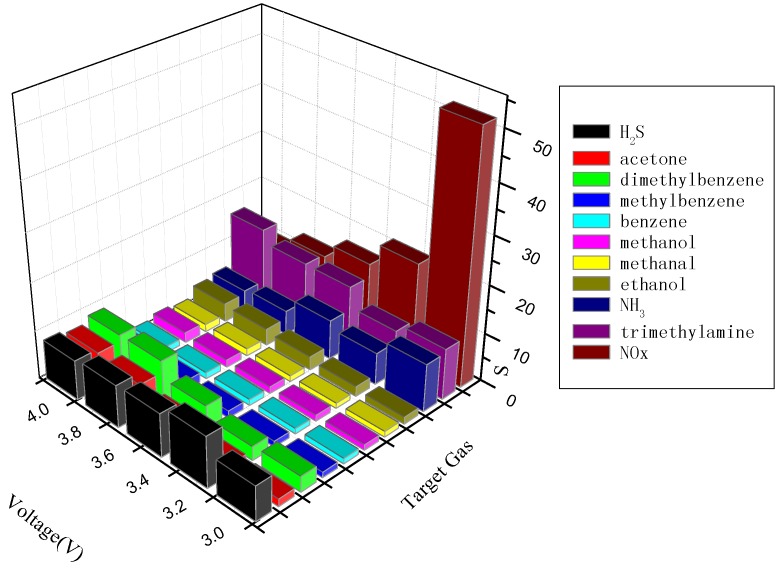
Responses of pure WO_3_ sensors to various gases at different operating voltages.

Selectivity is an important factor of gas sensors, so the responses of pure WO_3_ sensors to different kinds of target gases were measured at different operating voltages. The results are shown in Figure 8. It can be seen that the pure WO_3_ sensors exhibit the largest response to NO_x_ among all the tested gases at an operating voltage of 3.00 V. Furthermore, the optimal operating voltages are 3.00 V for NH_3_, 3.25 V for H_2_S and 4.00 V for acetone, dimethylbenzene, ethanol and trimethylamine. The response of the pure WO_3_ sensor to other target gases is extremely low at the tested operating voltage. As a result, the pure WO_3_ sensor is a very promising semiconductor for monitoring NO_x_ at relatively low temperatures, but its high resistance limited its wider application.

**Figure 9:**
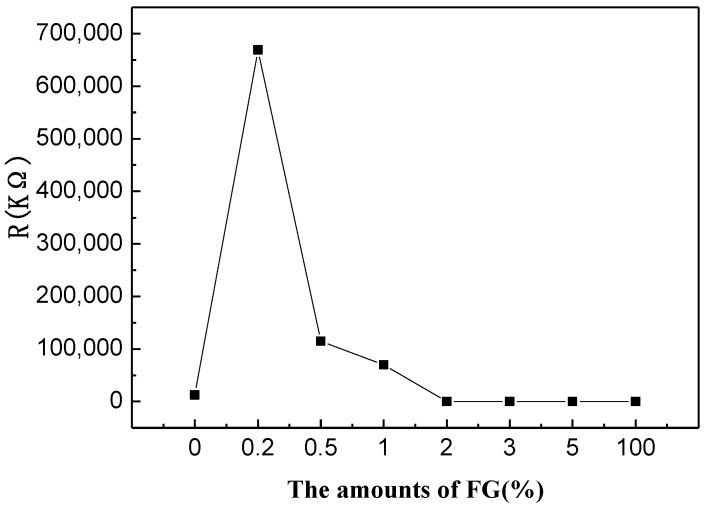
Resistances of the FG/WO_3_ nanocomposites with different amount of FG.

Resistances of the FG/WO_3_-nanocomposite-based sensors with different amounts of FG (0%, 0.2%, 0.5%, 1%, 2%, 3%, 5%, and 100%) are shown in Figure 9. It was clear that the resistance of the FG/WO_3_ nanocomposites with FG amount below 2% (higher than 69,644 kΩ) is noticeably higher than that of the pure WO_3_ sensor (12,501 kΩ). The sensors with few-layer graphene nanosheets and graphene/WO_3_ nanocomposites with graphene content higher than 1% have lower resistivity (below 135 kΩ) than that of the pure WO_3_ sensor. As we all know, WO_3_ is an n-type semiconductor and reduced graphene oxide is a p-type semiconductor, so it can form a p-n heterojunction between FG and WO_3_. The p-n heterojunction and the conduction of graphene play an important role in the resistance of FG/WO_3_ nanocomposites. As the amount of FG drops below 2%, the resistance of the FG/WO_3_ nanocomposites becomes controlled by the p-n heterojunction, so the resistance is really higher than that of WO_3_ and the sensors based on these are not suited for gas sensor application.

**Figure 10:**
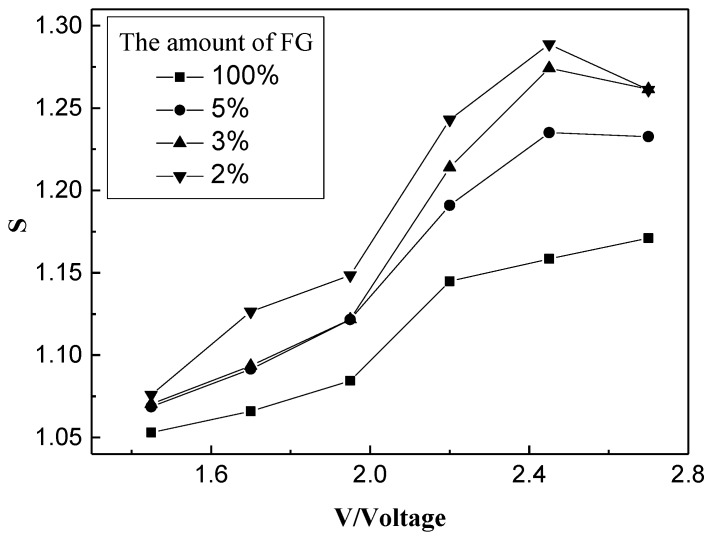
Responses of FG/WO_3_ nanocomposites sensors with different amounts of FG to 100 ppm NO_x_ at different operating voltage.

Gas sensitivity of the FG/WO_3_ nanocomposites was studied at an operating voltage between 1.45 and 2.70 V. It was found that FG/WO_3_ nanocomposites only had gas sensitivity to NO_x_. The responses of FG/WO_3_ nanocomposite sensors with different amounts of FG to 100 ppm NO_x_ at different operating voltages between 1.45 and 2.70 V are presented in Figure 10. Obviously, the sensitivity of the sensors to 100 ppm NO_x_ increased as the amount of FG decreased. Moreover, at operating voltages between 1.60 and 2.45 V, with the increase of operating voltage, the response of the FG/WO_3_ nanocomposite sensors was enhanced, and for operating voltages higher than 2.45 V, the response declined. As is well known, WO_3_ is very sensitive to NO_x_ at relative higher operating voltage. As the operating voltage was increased to 2.45 V, the effect of WO_3_ on the response of the FG/WO_3_ nanocomposite sensors to NO_x_ was enhanced. In other words, the counterbalance of the p-n heterojunction in the response of the FG/WO_3_ nanocomposite sensors to NO_x_ cannot be ignored, so the response to NO_x_ trends downward. Compared with the pure WO_3_ sensor, the sensitivity of FG/WO_3_ nanocomposite sensors was decreased, but both the resistance of the FG/WO_3_ nanocomposite sensors and the operating voltage were obviously lower.

**Figure 11:**
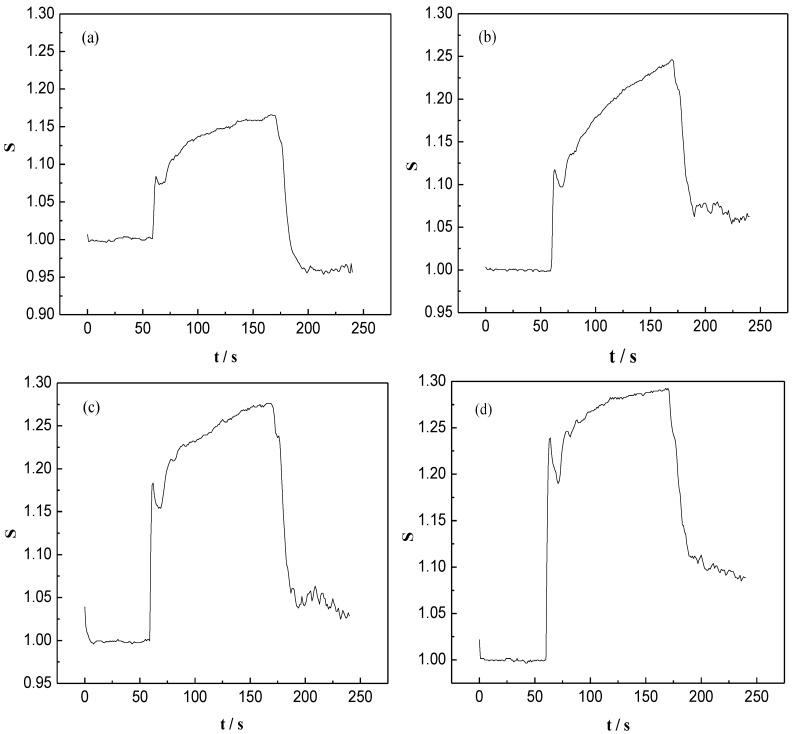
Responses of (**a**) FG, (**b**) FG (5%)/WO_3_ nanocomposites, (**c**) FG (3%)/WO_3_ nanocomposites and (**d**) FG (2%)/WO_3_ nanocomposites to 100 ppm NO_x_ at the operating voltage of 2.45 V.

The responses of FG, FG (5%)/WO_3_ nanocomposites, FG (3%)/WO_3_ nanocomposites, and FG (2%)/WO_3_ nanocomposites to 100 ppm NO_x_ at an operating voltage of 2.45 V are shown in Figure 11. The vertical coordinates were defined as *S* = *R*_g_/*R*_a_, where *R*_a_ is a fixed value. The strong increase in R_g_ of the sensors upon exposure to 100 ppm NO_x_ of the FG/WO_3_ nanocomposite sensors can be attributed to charge transfer between NO_x_ molecules and the sensing material, where NO_x_ act as an acceptor. It was clear that the response curves ascend or descend sharply with the inflow or outflow of NO_x_ gas, indicating that the sensors have an excellent response-recovery property.

## 3. Experimental Section

### 3.1. Synthesis of Graphite Oxide

All the reagents used in the experiments were in analytic grade and used without further purification. Natural graphite, hydrogen peroxide, and ammonium persulfate were mixed at a weight ratio of 2:1:0.1 in a beaker at room temperature with ultrasonication for 5 min. Subsequently, the mixture was transferred to a crucible, and then it was placed in a microwave oven and irradiated at 500 W for 90 s [26]. Under microwave irradiation, the precursors exfoliated rapidly, accompanied with lightening, then the mixture was dispersed with 3 M HCl aqueous solution (300 mL) with ultrasonication for 30 min and the suspension was allowed to stand at room temperature for another 24 h. Finally, the mixture was filtered and washed with 3 M HCl and distilled water for several times, respectively. After drying at 50 °C, the first exploited graphite (EG) was obtained.

Graphite oxides (GOs) were synthesized from EG using a modified Hummers method. In a typical procedure, 2.0 g of EG powder was put into 100 mL of cold (0 °C) concentrated H_2_SO_4_ (98%). Then, KMnO_4_ (8.0 g) was added gradually under stirring and the temperature of the mixture was kept to below 10 °C by cooling. The reaction was maintained for 2 h below 10 °C. Then, the ice bath was removed, and the reaction mixture was stirred at 35 °C for 1 h, and diluted with 100 mL of distilled water. Because the addition of water in concentrated H_2_SO_4_ medium released a large amount of heat, the addition of water was performed to keep the temperature below 100 °C. After adding 100 mL of distilled water, the mixture was stirred for 1 h at 90 °C and further diluted to approximately 300 mL with distilled water. After that, 20 mL of 30% H_2_O_2_ was added to the mixture to reduce the residual KMnO_4_. Finally, the mixture was filtered and redispersed with 1 M HCl aqueous solution (300 mL) and left overnight. Several sets of filtering and redispersal processes were carried out to wash the SO_4_^2−^ completely. The resulting solid was dried at 50 °C for 24 h.

### 3.2. Synthesis of Graphene/WO_3_ Nanocomposites

Graphite oxide was dispersed in 50 mL of distilled water with ultrasonication for 5 h (the second step of exfoliation of graphite), and the FGO solution was isolated by centrifugation, then 50 mL of ethanol was added into the FGO solution, and the pH value was adjusted to 8.0 with aqueous ammonia. One hundred and sixty mg of tungsten hexachloride (WCl_6_) was dispersed in the mixture solution with continued stirring for 24 h. Then, the solution was transferred into a Teflon-line autoclave, which was put in an oven of 200 °C for 10 h. After the autoclave was naturally cooled to room temperature, the as-synthesized products were isolated by centrifugation, washed several times with deionized water and absolute ethanol to remove Cl^−^, respectively, and finally dried in a vacuum oven at 70 °C for 24 h. In addition, FG and WO_3_ were synthesized in the same way as the composites in the absence of WCl_6_ and GO, respectively.

### 3.3. Material Characterization

Morphology of the products obtained was observed by using a JEOL JSM-7100F high-resolution thermal field emission scanning electron microscope (JEOL JSM-7100F FE-SEM, JEOL Ltd., Tokyo Japan) and a JEOL JEM-2100 (UHR) high-resolution transmission electron microscope (JEOL JEM-2100 (UHR) HRTEM). UV-vis spectroscopy measurements were performed on a UNICO UV-4802 UV-vis double-beam spectrophotometer (UNICO, Shanghai, China) in ethanol dispersion. Raman spectra were recorded on a Renishaw inVia-Reflex confocal Raman microscope (Renishaw, Shanghai, China) with 532 nm laser excitation. Crystalline structure and crystallite size of the samples were characterized by x-ray diffraction (XRD, Bruker D8, Rheinstetten, Germany), Cu Kα (λ = 0.15418 nm) radiation at 40 kV and 60 mA at room temperature. The TG-DTA measurement was carried out with a ZRY-1 instrument (Jiangdong, Suzhou, China) at a heating rate of 15 °C/min with air and N_2_ as the buffer gas.

### 3.4. Gas-Sensing Test

The fabrication process of gas sensors made from the as-prepared powder samples can be seen in [27]. The gas-sensing test was performed in a HW-30A (Hanwei Electronic Co. Ltd., Zhengzhou, China) measuring system. Electrical resistances in air and gas sensitivity were measured in the static state. The operating temperature of a sensor can be adjusted by varying the operating voltage. The tested operating voltages in this study are 1.45, 1.70, 1.95, 2.20, 2.45, 2.70, 3.00, 3.25, 3.50, 3.75 and 4.00 V, corresponding to operating temperatures of 60, 80, 85, 90, 100, 130, 160, 180, 200, 225 and 250 °C. In this paper, gas sensitivity was defined as *S* = *R*_a_/*R*_g_ to the reducing gas and *S* = *R*_g_/*R*_a_ to the oxidizing gas, where *R*_a_ and *R*_g_ are the resistances of a sensor in air and in a test gas, respectively.

## 4. Conclusions

Graphene oxides were prepared by using a two-step exploitation method, and few-layer graphene nanosheets and graphene/WO_3_ nanocomposites were prepared via a solvothermal method in an ethanol-distilled water medium. It was found that the obtained few-layer graphene nanosheets have the intrinsic nature of graphene nanosheets. The preferred growth direction of WO_3_ was changed in the graphene/WO_3_ nanocomposites. The morphology of graphene/WO_3_ nanocomposites appeared as a sandwich-like structure. The few-layer graphene nanosheets and graphene/WO_3_ nanocomposite sensors with graphene content higher than 1% have obviously lower resistivity (below 135 kΩ) than that of a pure WO_3_ sensor (12,501 kΩ), and they have the highest response to NO_x_ at a relatively lower operating voltage (2.45 V).
